# Initial observations on dose optimization in ^125^I seed implantation for recurrent glioblastoma: safety and efficacy across low- and high-dose ranges

**DOI:** 10.3389/fonc.2026.1867715

**Published:** 2026-07-13

**Authors:** Chengli Li, Ming Liu, Yujun Xu, Jing Fang, Wenjing Sun, Xingchang Yan, Yongquan Cao, Xiangmeng He

**Affiliations:** 1 Department of Interventional MRI, Shandong Provincial Hospital Affiliated to Shandong First Medical University, Jinan, China; 2 Department of Hemodialysis, The Second Affiliated Hospital of Shandong University of Traditional Chinese Medicine, Jinan, China; 3 Department of MRI, Cangzhou People’s Hospital, Cangzhou, China; 4 Department of Oncology, Clinical Medical College & Affiliated Hospital of Chengdu University, Chengdu, China; 5 Department of Medical Imaging, Zibo First Hospital, Zibo, China

**Keywords:** brachytherapy, doses, glioblastoma, iodine radioisotopes, magnetic resonance imaging

## Abstract

**Objective:**

To compare the efficacy and safety of low-dose versus high-dose iodine-125 (^125^I) seed implantation in patients with recurrent glioblastoma.

**Methods:**

This study retrospectively analyzed 62 patients with recurrent glioblastoma who underwent magnetic resonance (MR) combined with three-dimensional printed template-guided ¹²^5^I seed implantation between 2017 and 2024. Based on the prescribed dose, patients were stratified into two groups: 120–140 Gy and 140–160 Gy. The 6-month objective response rate (ORR), 1-year survival rate, median overall survival (OS) measured from the time of brachytherapy, and complications were compared between the two groups.

**Results:**

The 6-month ORR was 61.9% in the 120–140 Gy group and 73.2% in the 140–160 Gy group, with no statistically significant difference between the two groups (P = 0.362). The 1-year survival rate was 42.9% and 68.3% in the low- and high-dose groups, respectively (P = 0.053). Median OS was statistically longer in the high-dose group (13.7 months vs. 11.2 months, P = 0.001). Symptomatic brain edema occurred in 2 cases (Grade 2) in the low-dose group, and 10 cases in the high-dose group (7 Grade 2, 3 Grade 3). All 12 patients received corticosteroid and mannitol therapy, resulting in significant alleviation of clinical symptoms.

**Conclusion:**

¹²^5^I seed implantation represents a salvage treatment option for recurrent glioblastoma. Our retrospective data provide preliminary evidence that a higher dose (140–160 Gy) was associated with improved clinical outcomes compared to the lower dose range. These findings are hypothesis-generating and warrant validation in prospective randomized studies. Although higher doses increased the risk of postoperative brain edema, these complications were controllable with medical intervention.

## Introduction

The management of glioblastoma continues to pose a substantial clinical challenge. Despite multimodal standard therapy—including surgery, radiotherapy, and temozolomide chemotherapy—nearly all patients eventually experience recurrence, with a two-year mortality rate exceeding 90% (1). Glioblastoma is associated with a median OS of less than 15 months (2). Therapeutic options for recurrent glioblastoma are particularly limited and often ineffective. ^125^I seed implantation is a locoregional treatment modality that has been evaluated for the management of recurrent glioblastoma, with studies indicating its promising efficacy and acceptable safety (3–7). The recent development of three-dimensional (3D) template-assisted technology has improved the implantation accuracy and clinical efficacy of ¹²^5^I seed brachytherapy, warranting further in-depth investigation (8, 9).

A broad range of prescription doses (50–160 Gy) has been employed in prior studies of ¹²^5^I seed implantation for glioma, resulting in variability in clinical outcomes and complication rates (3–5, 10–13). When the clinical target volume (CTV) and seed dose rate are held constant, an optimal prescription dose range that maximizes therapeutic efficacy is expected to exist. Establishing a reasonable prescription dose range for recurrent glioblastoma is therefore of significant clinical relevance. This retrospective study aimed to compare clinical outcomes and complication profiles between lower-dose (120–140 Gy) and higher-dose (140–160 Gy) ¹²^5^I seed implantation regimens in patients with recurrent glioblastoma treated under MR and 3D-printed template guidance.

## Materials and methods

### Patients

A total of 62 patients with recurrent glioblastoma with 65 lesions underwent MR-guided 3D -printed template-assisted ¹²^5^I seed implantation between August 2017 and December 2024. According to the prescribed dose, patients were stratified into two groups: 120–140 Gy and 140–160 Gy. Patients treated with 120–140 Gy were enrolled consecutively between July 2018 and April 2020 (n=21). For the remainder of the study period, all patients were treated with 140–160 Gy (n=41). All patients had previously received surgery or radiochemotherapy prior to brachytherapy. Tumor recurrence was confirmed in 57 cases based on the Response Assessment in Neuro-Oncology (RANO) criteria, incorporating conventional contrast-enhanced MRI findings, clinical deterioration, and follow-up confirmation of progressive lesion enlargement. Among these 57 patients, 13 with atypical imaging features underwent additional functional imaging—including perfusion-weighted MRI and MR spectroscopy—to assist in the differential diagnosis. Histological confirmation was obtained by MRI-guided brain biopsy in the remaining 5 cases. The median age of patients was 58.5 years (range: 36–77 years), with a median tumor diameter of 3.6 cm (range: 1.6–8.5 cm). Baseline characteristics of the patients are summarized in **Table 1**, showing no statistically significant differences (p > 0.05) between the two dose groups.

**Table 1:** Patient characteristics by different prescribed dose groups.

Characteristic	120–140 Gy (n = 21)	140–160 Gy (n = 41)	Total (n = 62)	P value
Sex (n)				0.469
Male	11	25	36	
Female	10	16	26	
KPS^*^	50(40–90)	50(40–80)	50 (40–90)	0.969
Age (years) ^*^	59(40–74)	58(36–77)	58.5 (36–77)	0.954
Tumor diameter (cm) ^*^	3.9(1.6–8.5)	3.3(2.1–7.2)	3.6(1.6–8.5)	0.343
Tumor location				0.965
Frontal lobe	6	12	18	
Parietal lobe	7	13	20	
Occipital lobe	2	6	8	
Temporal lobe	6	13	19	
Previous treatment				0.680
S+R+T	18	37	55	
R+T	3	4	7	
MGMT status				0.678
Methylated	6	11	17	
Unmethylated	7	17	24	
Unknown	8	13	21	
IDH1 mutation				1.000
Mutated	1	2	3	
Wild type	11	24	35	
Unknown	9	15	24	

S, surgery; R, radiotherapy; T, temozolomide. ^*^ = median (range).

The inclusion criteria were as follows: (1) patients with glioblastoma who had received prior treatment comprising surgery and/or radiotherapy with temozolomide; (2) radiographic or histopathologic confirmation of disease recurrence or progression; (3) the number of tumor lesions ≤2; and (4) a life expectancy exceeding 2 months. Exclusion criteria comprised: (1) diffusely infiltrative tumors; (2) severe coagulation dysfunction; (3) impaired consciousness precluding patient cooperation; (4) any contraindication to MRI.

This study was performed in line with the principles of the Declaration of Helsinki. Approval was granted by the Ethics Committee of Shandong Provincial Hospital Affiliated to Shandong University (Permission: 2017-058). Informed consent was obtained from all individual participants included in the study.

### Equipment and technique

Brachytherapy was performed using ¹²^5^I radioactive seeds (Xinke Pharmaceutical Co., Ltd., Shanghai, China; Beijing Atom High-Tech Co., Ltd., Beijing, China) with an initial dose rate of 7.7 cGy/h and a half-life of 59.4 days. Preoperative planning was performed utilizing a brachytherapy treatment planning system (BTPS; Beijing University of Aeronautics and Astronautics, Beijing, China). The implantation procedure was guided with a 1.0-Tesla open MR system (Philips Healthcare, Amsterdam, The Netherlands) combined with a patient-specific 3D-printed template (Zhuoye Electronic Technology Co., Ltd., Zibo, China). Puncture and seed implantation were conducted using MR-compatible 18-gauge blunt-tip needles. Skull penetration was achieved with high-speed drills (diameter: 1.9 mm).

### Pre-operative preparation

Prior to contrast-enhanced MRI scanning utilizing isovoxel T1-weighted imaging (1 mm slice thickness), fiducial markers (fish oil capsules) were placed on the patients’ scalps. The acquired T1-weighted images were then exported to the BTPS for preoperative planning. The gross tumor volume (GTV) was delineated as the contrast-enhanced tumor boundary. Based on previous studies, the clinical target volume (CTV) was defined by contouring a 10-mm isotropic expansion beyond the gross tumor volume (GTV) (3, 10, 14, 15). Dosimetric parameters, including D90 and V100, were calculated to assess dose distribution quality. Dose conformity was evaluated using the conformity index (CI), and the external index (EI) was used to quantify the volume of normal brain tissue receiving the prescription dose outside the CTV. The puncture trajectory, as well as the number, activity, and spatial distribution of the ¹²^5^I seeds, were determined. The puncture trajectory was designed to avoid blood vessels and dural sinuses. Based on the preoperative plan, a patient-specific, 3D-printed non-coplanar template was fabricated with a printing accuracy of 0.05 mm.

### Brachytherapy procedure

**Figure 1:**
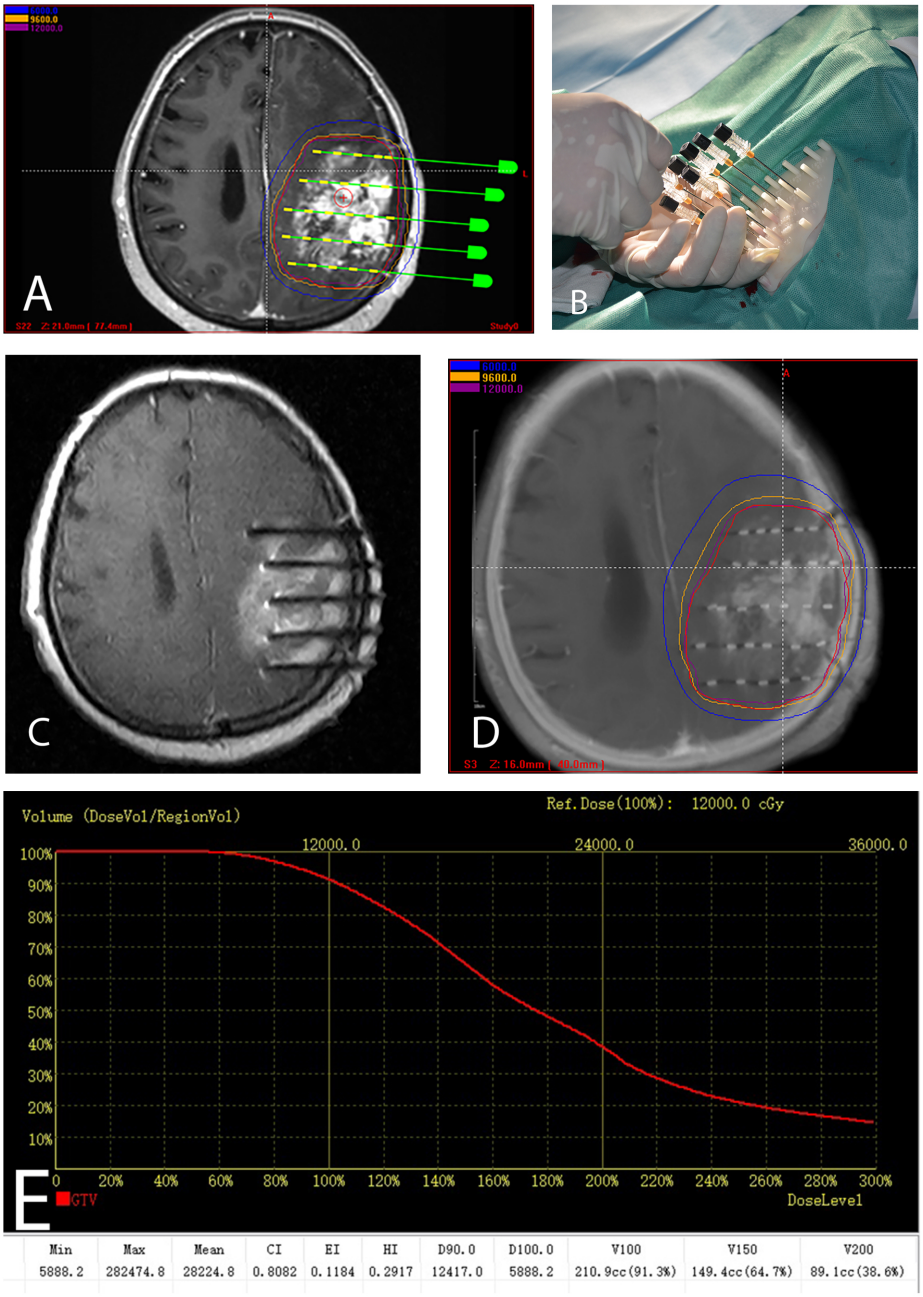
A patient with recurrent glioblastoma after surgery, radiotherapy, and temozolomide chemotherapy underwent ¹²^5^I seed implantation assisted by a 3D-printed template. **(A)** Pre-operative treatment plan. **(B)** Blunt-tip needles were inserted into the tumor through the puncturing template. **(C)** The positions of needles were visualized on axial MR images. **(D)** Post-operative dosimetry verification based on post-operative CT/MR fusion images. **(E)** Postoperative dose volume histogram (D90 = 124.2 Gy).

¹²^5^I seeds were implanted into the tumor under the guidance of a 1.0-T open MRI system combined with a 3D-printed template. The template was affixed to the scalp following alignment with predefined fiducial markers. Prior to the procedure, a T1-weighted turbo spin echo (T1W-TSE) sequence (3 mm slice thickness) was acquired to verify the precise positioning of the template. Following confirmation of template placement, the patient was temporarily moved out of the magnet bore. Local anesthesia was achieved with lidocaine (2%), and intravenous sedation was administered using diazepam. Cranial burr holes were created through the drilling guides of the template using a battery-operated twist drill. Blunt-tip needles were advanced into the tumor to the preoperatively planned depths under template guidance. The patient was subsequently repositioned inside the magnet bore for a confirmatory MRI scan to visualize the trajectories of the implanted needles (**Figure 1**). Finally, ¹²^5^I seeds were implanted directly through blunt-tip needles, without the use of an absorbable carrier material. Following the preoperative plan, seeds were deposited individually at the predetermined spacing as the needle was gradually withdrawn, with no cannula left indwelling in the tissue. A slow withdrawal rate was maintained throughout to avoid abrupt intratissue pressure fluctuations that could otherwise result in seed migration. The ^125^I seeds were intended as permanent interstitial implants and were not designed to be retrieved following placement.

### Post-operative dosimetry verification

Post-operative CT and MRI scans were acquired and co-registered to create fused CT/MR images. The fused images were transferred to the BTPS for post-operative dosimetry verification. Dose-volume histogram parameters, including D90, V100, CI, and EI, were calculated based on the fused image set.

### Clinical follow-up

All patients received no additional systemic therapy after ¹²^5^I seed implantation. Follow-up assessments consisted of contrast-enhanced MRI and clinical evaluation performed at 3-month intervals after brachytherapy. Treatment-related toxicities were graded according to the Radiation Therapy Oncology Group (RTOG)/European Organization for Research and Treatment of Cancer (EORTC) scoring system and the Common Terminology Criteria for Adverse Events (CTCAE, version 4.0). Treatment response was evaluated using the Response Assessment in Neuro-Oncology (RANO) criteria and classified as complete response (CR), partial response (PR), stable disease (SD), or progressive disease (PD).

### Statistical analysis

Dosimetric concordance between preoperative plans and postoperative verification was evaluated using paired t-tests or Wilcoxon signed-rank tests for matched comparisons. A comparative assessment of dosimetry parameters between the two dose cohorts (120–140 Gy and 140–160 Gy) was performed using Student’s t-test or the Wilcoxon rank-sum test. The objective response rate (ORR) at 6 months was defined as the proportion of patients who achieved a complete or partial response (CR + PR) among all evaluated patients within each prescribed dose cohort. The median overall survival (OS) measured from the date of ¹²^5^I seed implantation was estimated using the Kaplan-Meier method. Between-group differences in the 6-month ORR and 1-year survival rate were analyzed using the chi-square test, and survival curves were compared with the log-rank test.

Multivariable Cox proportional hazards regression analysis was performed to evaluate the association between D90 and overall survival, adjusting for age, KPS, and tumor size. The proportional hazards assumption was assessed using log-minus-log survival plots, which confirmed that the assumption was satisfied for all covariates. Spearman correlation analysis was conducted to assess the dose-response relationship. For all regression and correlation analyses, 3 patients with multiple lesions were excluded to ensure independence of observations, resulting in an analytical cohort of 59 patients. Statistical analyses were performed using SPSS 22.0 software, with P values < 0.05 considered statistically significant.

## Results

### Dosimetry verification

Dosimetric concordance between preoperative plans and postoperative verification was confirmed, with no significant differences in D90, V100, CI, or EI (all P > 0.05) (**Table 2**). The prescribed dose for the entire cohort ranged from 120 to 160 Gy, with a median of 145 Gy. The median postoperative D90 was 128.8 Gy (range: 114.6–139.2) in the 120–140 Gy group and 149.3 Gy (range: 140.0–179.4) in the 140–160 Gy group, a difference that was statistically significant (p <0.001). The mean postoperative V100 values were 91.7 ± 1.2% in the 120–140 Gy group and 92.2 ± 1.8% in the 140–160 Gy group. The median CI values were 0.76 and 0.75, while the median EI values were 21.7% and 23.2%, respectively. No significant differences in V100, CI, or EI were observed between the two groups (p > 0.05) (**Table 3**).

**Table 2:** Comparison of preoperative and postoperative dosimetry parameters.

Parameter	120–140 Gy		140–160 Gy			
	Preoperative	Postoperative	P*	Preoperative	Postoperative	P*
D90 (Gy)	129.2 (120.0–138.4)	128.8 (114.6–139.2)	0.465	150.0 (140.7–174.0)	149.3 (140.0–179.4)	0.546
V100 (%)	91.8 ± 1.2	91.7 ± 1.2	0.438	92.3 ± 1.5	92.2 ± 1.8	0.743
CI	0.76 (0.63–0.81)	0.76 (0.62–0.81)	0.265	0.75 (0.61–0.84)	0.75 (0.61–0.83)	0.442
EI (%)	21.1 (7.0–31.5)	21.8 (6.8–38.9)	0.223	22.0 (5.3–36.0)	23.2 (4.9–38.2)	0.272

Data are presented as median (range) for non-normally distributed variables and mean ± SD for normally distributed variables.

**Table 3:** Comparison of postoperative dosimetric parameters between the 120–140 Gy and 140–160 Gy groups.

Parameter	120–140 Gy group			140–160 Gy group			P value
	Range	Median	M (SD)	Range	Median	M (SD)	
D90 (Gy)	114.6–139.2	128.8	128.4(6.6)	140–179.4	149.3	153.2(10.4)	<0.001
V100 (%)	90.1–94.4	91.3	91.7 (1.2)	89.0–96.9	92.1	92.2 (1.8)	0.174
CI	0.62–0.81	0.76	0.74 (0.06)	0.61–0.83	0.75	0.74 (0.06)	0.744
EI (%)	6.8–38.9	21.7	21.8(8.9)	4.9–38.2	23.2	21.9(8.7)	0.901

M, mean; SD, standard deviation.

### Local control and survival

**Figure 2:**
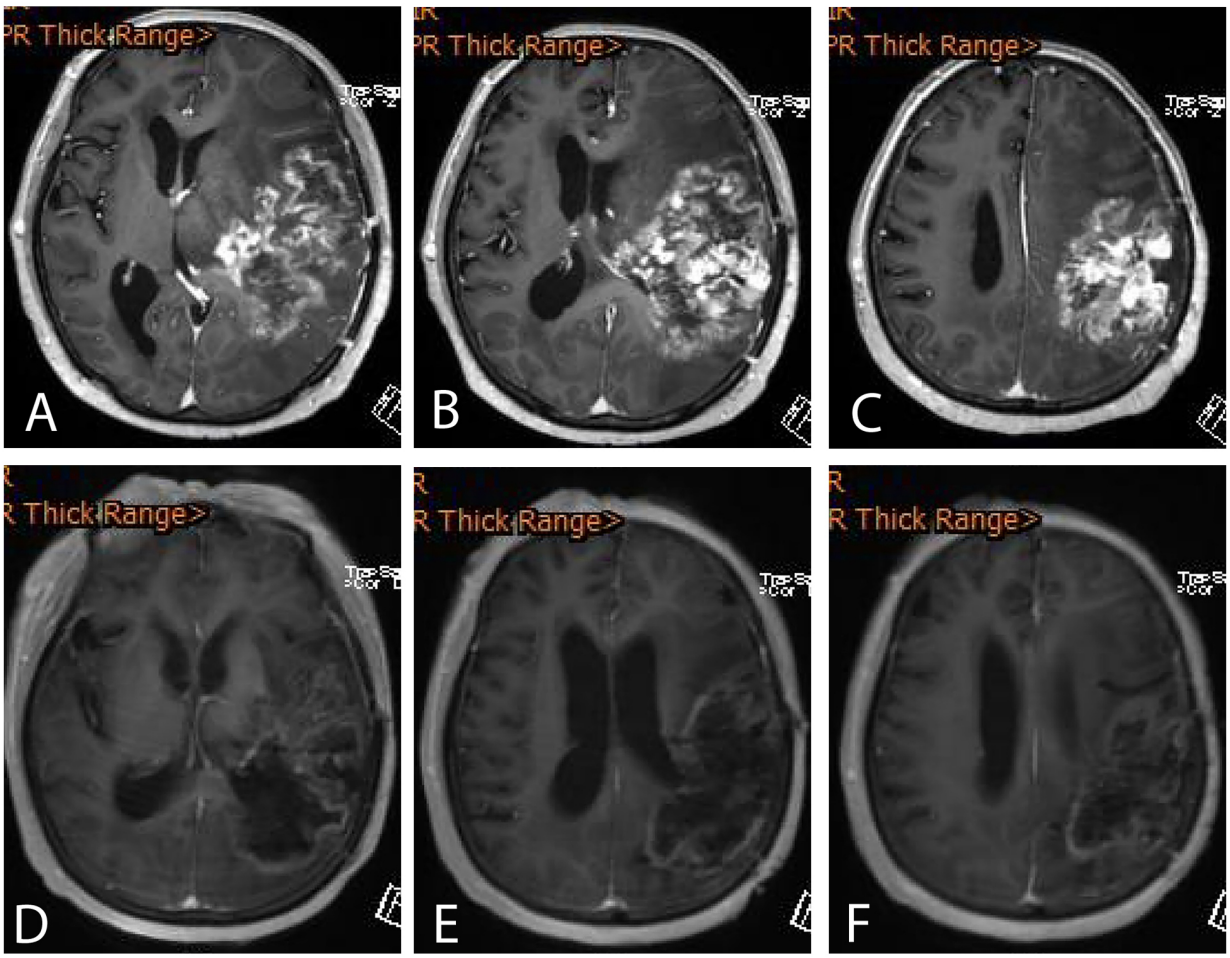
Postoperative follow-up of the case shown in **Figure 1** by MRI. **(A–C)** MR images before ^125^ I brachytherapy. **(D–F)** At 6-month follow-up, contrast-enhanced T1W images demonstrate marked decreases in tumor enhancement, edema, and midline shift.

**Figure 3:**
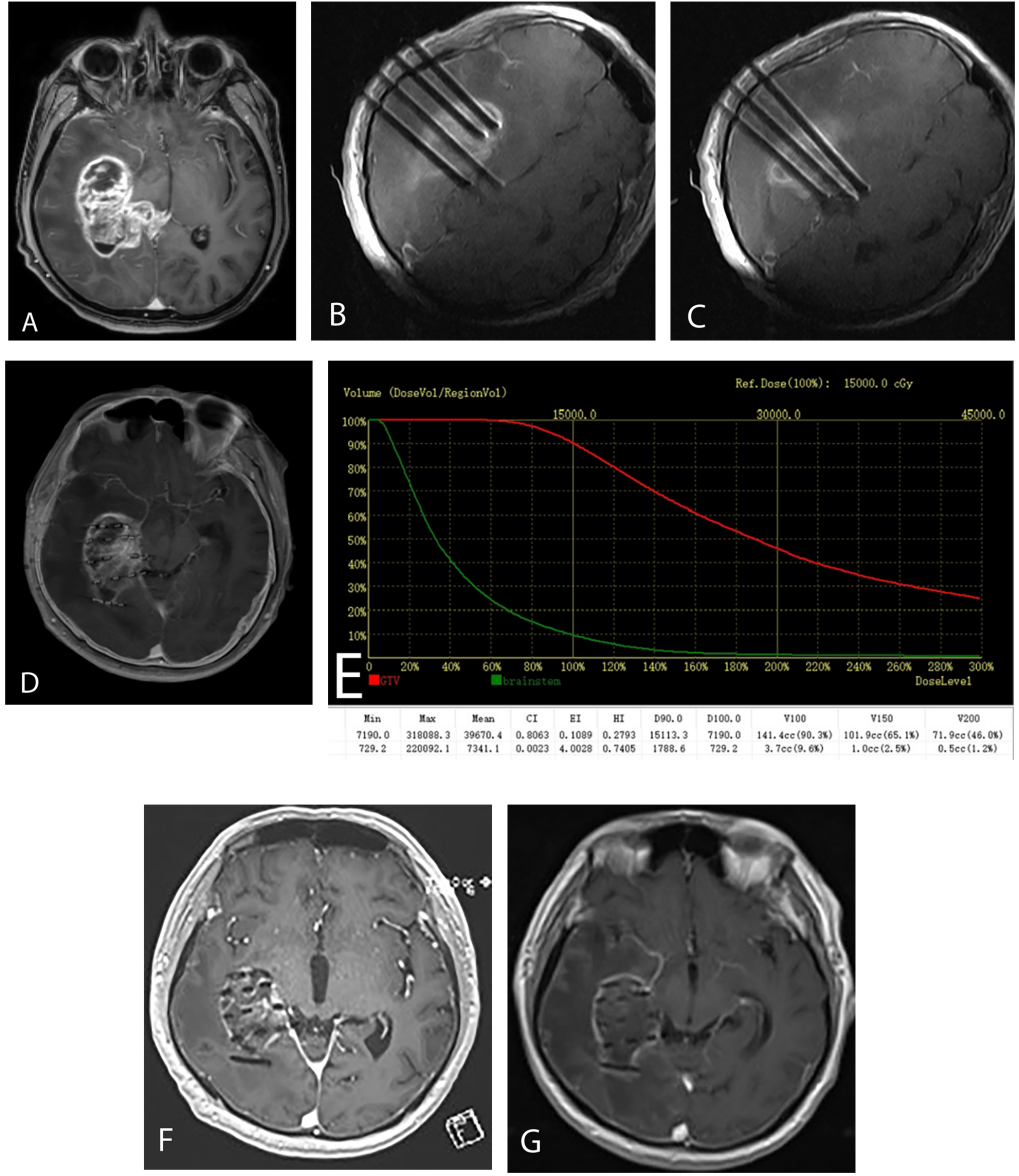
A case of glioblastoma progression after radiotherapy and temozolomide treatment receiving 3D-printed template-assisted ¹²^5^I brachytherapy. **(A)** Preoperative contrast-enhanced MRI showing tumor invasion into the right lateral ventricle. **(B, C)** Needles were inserted into the tumor through the template and displayed by MR images. **(D)** Post-operative CT/MR fusion images. **(E)** Postoperative dose volume histogram (D90 = 151.1 Gy). **(F, G)** Contrast-enhanced T1W images were obtained at 6 and 12 months after brachytherapy. These images demonstrate a significant reduction in tumor enhancement along with a marked decrease in mass effect, indicating effective local control.

**Figure 4:**
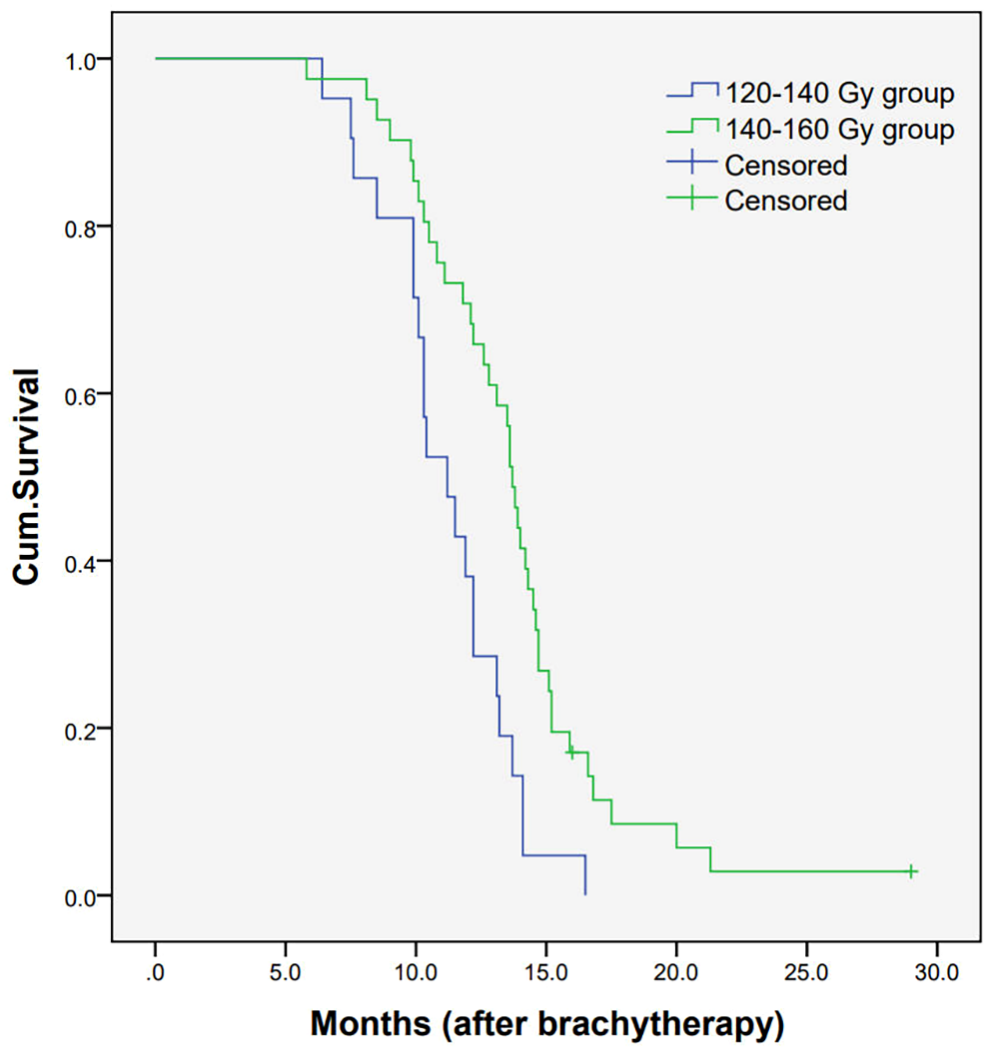
Kaplan-Meier plot for overall survival after ¹²^5^I brachytherapy in the two dose groups (120–140 Gy and 140–160 Gy).

The median follow-up time for the entire cohort was 13.0 months (range: 5.8–29.0 months). At the 6-month follow-up, treatment responses in the 120–140 Gy group were: complete response (CR) in 1 patient, partial response (PR) in 12, stable disease (SD) in 3, and progressive disease (PD) in 5 (**Figure 2**). In the 140–160 Gy group, responses consisted of CR in 2 patients, PR in 28, SD in 3, and PD in 8 (**Figure 3**). The ORR at 6 months was 61.9% (13/21) in the 120–140 Gy group and 73.2% (30/41) in the 140–160 Gy group, with no statistically significant difference between the groups (p = 0.362). The overall 1-year survival rate was 59.7% (37/62). Specifically, 1-year survival rates were 42.9% (9/21) for the 120–140 Gy group and 68.3% (28/41) for the 140–160 Gy group, although this difference was not statistically significant (P = 0.053). The median OS was 12.8 months for the entire cohort. Analysis by dose group revealed a median OS of 11.2 months in the 120–140 Gy group and 13.7 months in the 140–160 Gy group. There was a statistically significant difference (P = 0.001) between the two groups (**Figure 4**).

**Figure 5:**
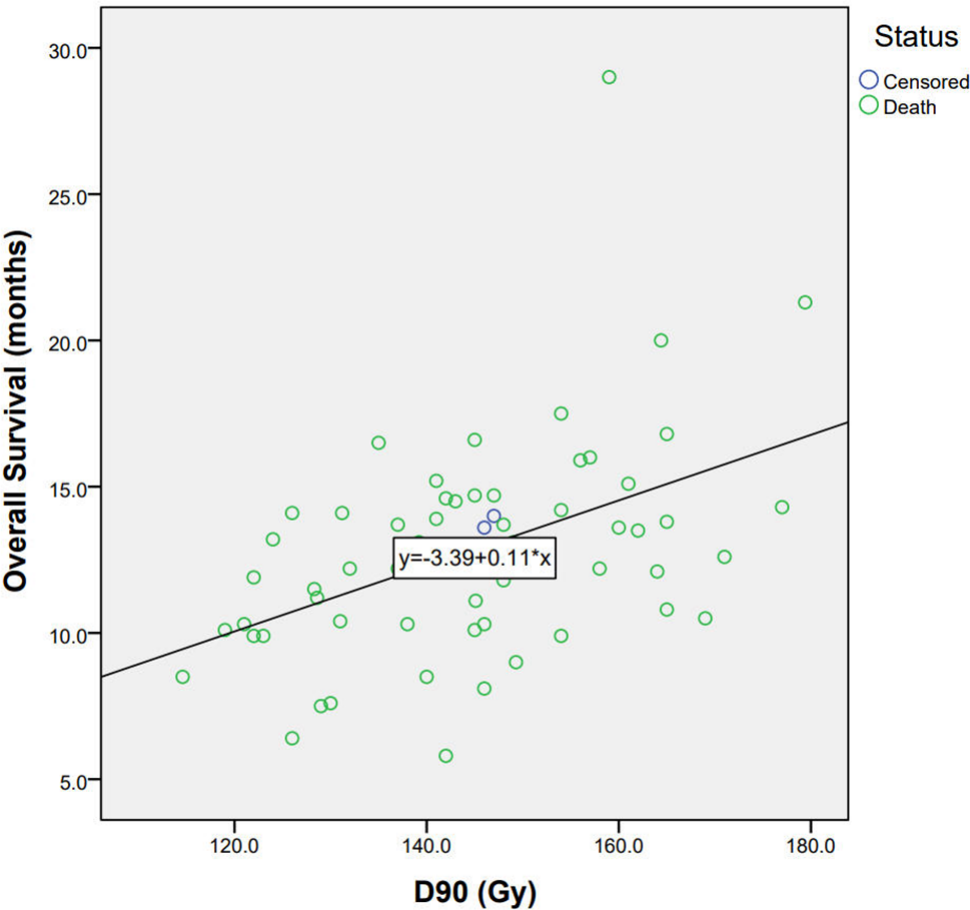
Scatter plot of D90 versus overall survival. Green circles indicate death, and blue circles represent censored patients. The solid line shows the linear regression fit (y = -3.39 + 0.11x). Spearman correlation analysis demonstrated a moderate positive correlation (r = 0.448, P < 0.001), confirming a dose-response relationship between D90 and overall survival.

On multivariable Cox regression, higher D90 was associated with improved OS (HR = 0.69 per 10 Gy, 95% CI 0.548–0.871, P = 0.003) (**Table 4**). Tumor size (HR = 1.29 per 1 cm, 95% CI 1.03–1.61, P = 0.027) and KPS (HR = 0.96 per point, 95% CI 0.93–0.99, P = 0.011) were also independent prognostic factors. Age was not significantly associated with OS (HR = 0.99 per year, 95% CI 0.96–1.02, P = 0.403). A scatter plot visualized the dose-response relationship between D90 and overall survival (**Figure 5**). Spearman correlation analysis further confirmed this association (r = 0.448, P < 0.001).

**Table 4:** Multivariable cox regression for overall survival.

Variable	HR (95% CI)	P value
D90 (per 10 Gy)	0.690 (0.548–0.871)	0.003
Tumor size (cm)	1.286 (1.029–1.609)	0.027
KPS	0.958 (0.927–0.990)	0.011
Age (years)	0.987 (0.957–1.018)	0.403

HR, hazard ratio; CI, confidence interval.

### Complication and toxicity

**Figure 6:**
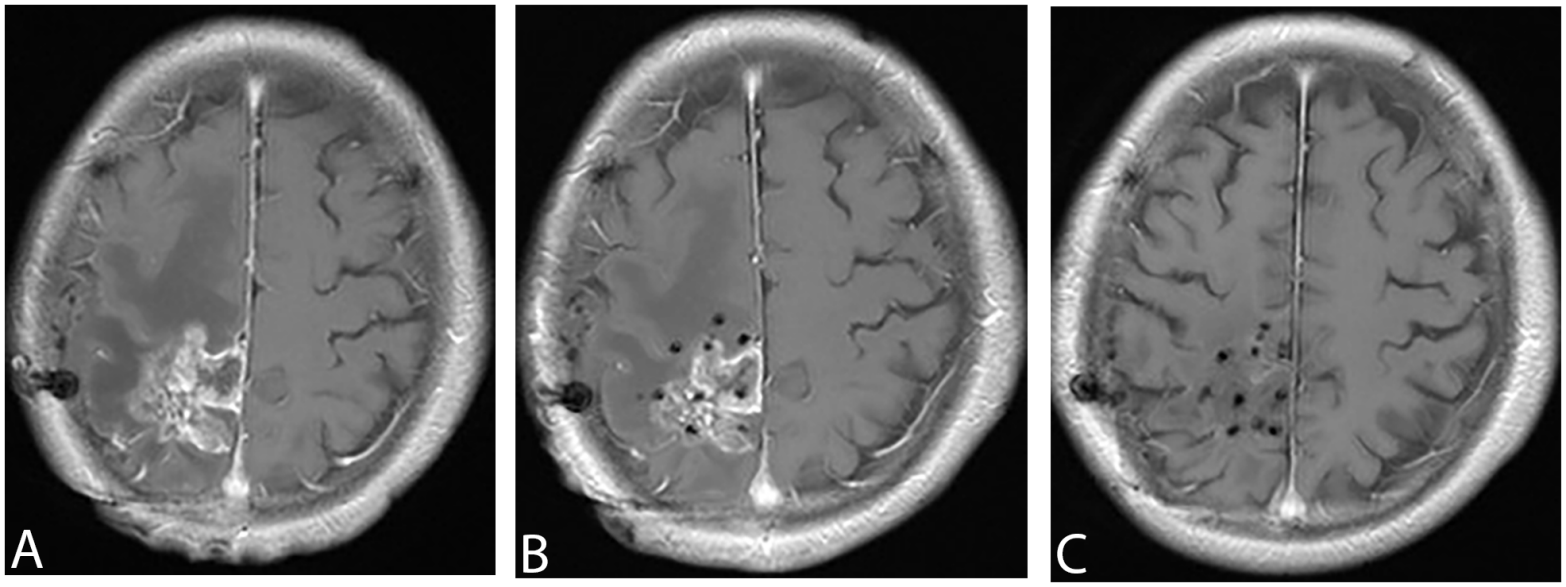
Symptomatic brain edema after ^125^I seed implantation in a patient with recurrent glioblastoma. **(A)** Preoperative contrast-enhanced MRI showing the tumor in the right parietal lobe with surrounding peritumoral edema. **(B)** One week after ¹²^5^I seed implantation, the patient developed headache and left hemiparesis. MRI confirmed postoperative brain edema. One-week treatment with mannitol and corticosteroids resulted in significant symptomatic relief. **(C)** There-month follow-up MRI showed marked reduction in tumor enhancement and significant alleviation of brain edema compared with baseline.

Each dose cohort (120–140 Gy and 140–160 Gy) experienced two cases of minimal hemorrhage. All hemorrhagic events were controlled using the hemostatic agent ethamsylate, with no bleeding progression observed within 24 hours post-treatment. No seed explantation was required in any case throughout the study period — no seeds were removed due to migration. Serial imaging follow-up revealed no evidence of significant seed migration. The incidence rate of brain edema was 9.5% (2/21) in the 120–140 Gy cohort and 24.4% (10/41) in the 140–160 Gy cohort, with no statistically significant difference between groups (P = 0.195). In the 120–140 Gy group, two patients developed symptomatic brain edema (Grade 2) after ¹²^5^I seed implantation. In the 140–160 Gy group, three and seven patients experienced Grade 3 and Grade 2 brain edema, respectively. The median time to onset was 2 weeks (range: 1-4) after implantation, and the median duration was 2.5 weeks (range: 2-4). Two patients in the high-dose group required hospitalization. All 12 affected patients received corticosteroid and mannitol therapy for 2–4 weeks, which led to significant symptomatic relief (**Figure 6**). Detailed characteristics are presented in **Table 5**.

**Table 5:** Characteristics of radiation-induced brain edema.

Characteristic	120–140 Gy (n = 2)	140–160 Gy (n = 10)	Total (N = 12)
Edema Grade			
Grade 2	2	7	9
Grade 3	0	3	3
Time to onset (weeks), median (range)	2 (1-3)	2 (1-4)	2 (1-4)
Duration (weeks), median (range)	2.5 (2-3)	2.5 (2-4)	2.5 (2-4)
Hospitalization required	0	2	2
Neurological symptoms			
Headache	2	6	8
Nausea/vomiting	1	2	3
Seizures	0	1	1
Motor deficit	0	2	2
Speech disturbance	1	0	1
Medications			
Mannitol	2	10	12
Corticosteroids	2	10	12
Antiepileptics	0	1	1

## Discussion

The current therapeutic approaches for recurrent glioblastoma primarily comprise reoperation, reirradiation, and chemotherapy. However, each modality is associated with inherent limitations. Although reoperation can confer a survival benefit in selected cases, its efficacy is contingent upon factors such as tumor location and the patient’s overall health status. Specifically, surgical resection of tumors located in eloquent brain areas carries a heightened risk of postoperative neurological deficits. The resectability of recurrent glioblastoma is often limited, precluding gross total resection in a significant number of cases (16). The efficacy of reirradiation in recurrent glioma is limited by the trade-off between delivering a tumoricidal dose and minimizing the risk of radiation-induced toxicity to the surrounding normal brain parenchyma. Systemic agents, including temozolomide and other alkylating agents, demonstrate limited efficacy in the recurrent setting, wherein acquired drug resistance remains a major clinical challenge (17).

Given these challenges, there is a clear rationale for exploring alternative or adjunctive local treatment strategies. Among these, the implantation of ^125^I radioactive seeds represents a minimally invasive interventional approach that has been investigated for recurrent gliomas. Previous studies have reported its potential in prolonging overall survival, supporting its consideration as a salvage treatment option (3, 18–20). Critically, and in contrast to external beam reirradiation, the rapid dose fall-off of ^125^I seeds (governed by the inverse square law) may allow for the delivery of a high focal dose to the tumor while sparing surrounding normal brain tissue. This physical characteristic makes it a particularly attractive option for patients who have previously received radiotherapy (21, 22).

This study demonstrated a median OS of 12.8 months following ¹²^5^I seed implantation for recurrent glioblastoma, which compares favorably to the previously reported median OS of 3–6 months, suggesting therapeutic benefit from this salvage modality (23, 24). In studies reporting ¹²^5^I seed implantation for tumors outside the central nervous system, the prescribed dose typically ranges from 100 to 145 Gy (25–28). Given that glioblastoma are notably radioresistant, there is a strong rationale for evaluating the clinical efficacy of dose escalation in this context. To our knowledge, no comparative studies have yet examined different prescribed dose levels in this patient population. In this study, the two dose cohorts (120–140 Gy and 140–160 Gy) were well-balanced with respect to baseline characteristics, including tumor size, Karnofsky Performance Status (KPS), age, prior treatment history, MGMT promoter methylation status, and IDH1 mutation status. The accurate seed implantation, validated by dosimetric concordance between preoperative plans and postoperative verification, was enabled by MR guidance with a 3D-printed template-assisted technique and ensured comparable postoperative dosimetric parameters (V100, CI, EI) between groups. Given the comparable prognostic profiles, clinical outcomes and complications were compared between the cohorts. The high-dose group (140–160 Gy) exhibited a statistically significantly longer median OS than the low-dose group (120–140 Gy) (13.7 months vs. 11.2 months, P = 0.001), suggesting that dose escalation within this range may confer a survival advantage. These findings highlight the potential of optimized dose strategies to improve clinical outcomes in recurrent glioblastoma patients receiving ¹²^5^I brachytherapy. The OS benefit observed in our study aligns with contemporary radiobiological perspectives, suggesting that tumors with diminished radiosensitivity such as recurrent glioblastoma may require higher biological effective doses to overcome their substantial self-repair capacity. The continuous low-dose-rate irradiation characteristic of ¹²^5^I seeds, potentially through inhibiting tumor cell repopulation and promoting apoptosis, may further amplify this dose-dependent effect (29).

It is important to note that this dose range corresponds to a CTV defined by a 10-mm margin around the gross tumor volume, and prescribed doses are not directly comparable when different CTV margins are applied. Further escalation of the prescribed dose was not pursued in this study due to the associated requirement for more implantation tracks, which would prolong surgical duration, increase procedural difficulty, and elevate the risk of surgical complications such as cerebral hemorrhage. Utilizing seeds with higher activity may represent a viable alternative for future dose-escalation approaches and potentially widening the therapeutic window. Furthermore, integrating molecular subtyping (e.g., IDH mutation and MGMT promoter methylation status) into prognostic models and dose selection criteria represents a critical step toward achieving individualized dose prescription.

Notably, dose-escalation studies in external beam radiotherapy (EBRT) have not demonstrated a survival benefit for glioblastoma, which differs from the findings of our study (30). This discrepancy arises from key radiobiological and technical distinctions between interstitial brachytherapy and EBRT. First, ¹²^5^I seed implantation exhibits a steep dose gradient, enabling high-dose delivery to the GTV with relative sparing of normal tissue. EBRT, characterized by a more homogeneous dose distribution, represents a differing dosimetric approach where escalation is limited by normal tissue constraints. Second, ¹²^5^I seeds deliver continuous, low-dose-rate irradiation throughout their radioactive decay period. This protracted exposure effectively suppresses tumor cell repopulation and impairs the repair of sublethal radiation damage. The effective local control achieved within the brachytherapy target volume underlies the survival benefit. This is critical for recurrent glioblastoma, considering that the disease is driven mainly by local progression rather than distant metastasis. Nevertheless, this approach shares an inherent constraint with other local modalities: infiltrative tumor cells beyond the predefined target volume may not receive a therapeutic dose due to the physical attenuation of radiation. Consequently, optimal local control within the target volume is a major contributor to, but does not solely determine, the overall survival outcome. Future strategies combining maximal local control with effective systemic or regional therapies targeting microscopic lesions may hold the key to further improving outcomes.

In this study, all patients with recurrent glioblastoma had previously been treated with radiotherapy before receiving ¹²^5^I seed implantation, a re-irradiation setting generally considered to elevate the risk of brain toxicity (31). This study observed such a complication, with an overall postoperative symptomatic brain edema incidence of 19.4%. Furthermore, differences in both the incidence and severity of brain edema were noted across dose groups. In the present cohort, the high-dose group (140–160 Gy) showed an increase in both the frequency and severity of this complication relative to the low-dose group (120–140 Gy). Comparative analysis revealed a numerically higher rate of symptomatic brain edema in the high-dose group (24.4% vs. 9.5% in the low-dose group). Although this difference did not reach statistical significance (P = 0.195), the more than a two-fold increase in incidence points to a potential dose-dependent trend—one that may hold clinical relevance and thereby supports a dose-dependent risk profile for this intervention. Nevertheless, the clinical symptoms related to brain edema were effectively managed medically, with substantial resolution achieved in all affected cases. These findings highlight the importance of postoperative monitoring, assessment, and timely therapeutic management, and further demonstrate that the associated toxicity is predictable, manageable, and reversible. No radiation-induced cerebral necrosis was observed in this study aside from cerebral edema, which can be attributed to three key factors. First, the use of 3D-printed template-assisted technique improved dose distribution conformity, thereby reducing radiation-induced damage to normal brain tissues (3). Second, the characteristic dose attenuation of ¹²^5^I seeds created a steep dose gradient from the center to the periphery of the target volume, which effectively limited radiation exposure to surrounding tissues. Third, the low-dose-rate characteristic of ¹²^5^I seeds allowed for more effective repair of sublethal damage in the peri-target normal brain tissue over the prolonged irradiation period.

This study has several limitations. First, the single-center and non-randomized design may introduce selection bias. Dose stratification by study time period carries potential for temporal confounding. However, all procedures were performed by the same team using a standardized template-assisted technique, and only cases from the standardized phase were included, which minimized temporal variability. Multivariable Cox regression demonstrated that D90 remained significantly associated with overall survival (HR = 0.69 per 10 Gy, P = 0.003). Second, no correction for multiple comparisons was applied, as overall survival was the primary endpoint. The secondary analyses (ORR and 1-year survival) should therefore be interpreted as exploratory. Third, molecular characterization was incomplete: MGMT promoter methylation status was not assessed in 21 of 62 patients (33.9%) and IDH1 mutation status in 24 of 62 patients (38.7%), limiting the ability to fully adjust for these prognostic confounders. Despite these limitations, our findings provide evidence supporting a dose-response relationship between D90 and survival, warranting validation in prospective randomized studies.

In summary, ^¹²^5^^I seed brachytherapy is an effective salvage therapy for recurrent glioblastoma. In this retrospective study, the high-dose cohort (140–160 Gy) was associated with improved overall survival compared to the lower-dose group (120–140 Gy). These findings are hypothesis-generating and warrant validation in prospective randomized studies. Although this regimen was associated with a higher incidence of symptomatic brain edema, these adverse events were effectively controlled with medication, suggesting a manageable risk-benefit profile.

## Data Availability

The original contributions presented in the study are included in the article/supplementary material. Further inquiries can be directed to the corresponding author.
